# Influence of chronic alcohol consumption on cerebral ischemia/reperfusion injury in female mice

**DOI:** 10.3389/fncel.2025.1600725

**Published:** 2025-06-04

**Authors:** Utsab Subedi, Pushpa Subedi, Asia Rogers, Xiao-Hong Lu, Manikandan Panchatcharam, Hong Sun

**Affiliations:** ^1^Department of Cellular Biology and Anatomy, LSU Health Shreveport, Shreveport, LA, United States; ^2^Department of Pharmacology, Toxicology and Neuroscience, LSU Health Shreveport, Shreveport, LA, United States

**Keywords:** alcohol, female, angiogenesis, ischemic stroke, inflammation

## Abstract

Light alcohol consumption (LAC) protects against cerebral ischemia/reperfusion (I/R) injury, whereas heavy alcohol consumption (HAC) worsens it in male mice. The phenomenon appeared to be associated with the dose-dependent influence of alcohol on cerebral angiogenesis and post-ischemic inflammation. However, whether there is a sex-specific difference is unknown. Therefore, the goal of this study was to examine the influence of chronic alcohol consumption on cerebral I/R injury in female mice. Female C57BL/6J mice were gavage-fed with 0.7 g/kg/day ethanol (designed as LAC), 2.8 g/kg/day ethanol (designed as HAC), or volume-matched water (designed as control) for 8 weeks. Subsequently, they were subjected to unilateral middle cerebral artery occlusion (MCAO) for 60 min. Under basal conditions, LAC reduced erythrocytes, whereas HAC reduced lymphocytes and monocytes. Neither LAC nor HAC affected exploratory behavior and memory performance, but both improved spontaneous motor activity and reduced anxiety. In addition, both LAC and HAC upregulated VEGFR2 and promoted cerebral angiogenesis. Furthermore, LAC upregulated TGF-β and TGF-βR2 and HAC upregulated VEGF-A. Following MCAO, LAC significantly reduced cerebral I/R injury, blood-brain barrier (BBB) disruption, neutrophil infiltration, and microglial activation and increased cerebral angiogenesis at 72 h of reperfusion. In contrast, although HAC reduced BBB disruption and neutrophil infiltration, it did not significantly alter cerebral I/R injury, post-ischemic cerebral angiogenesis, or microglial activation. Our findings suggest that LAC protects against transient focal cerebral ischemia in female mice. The beneficial effect may be related to its pro-angiogenic and anti-inflammatory properties.

## Introduction

Stroke, which 87% of cases are ischemic stroke, is a leading cause of death and disability worldwide (Hernández et al., 2021; Hou et al., 2024). Ischemic stroke occurs when blood flow to the brain is reduced or blocked due to a narrowed or obstructed artery (Hui et al., 2024). Since the brain highly demands a constant supply of oxygen and nutrients through blood flow, a slightly prolonged ischemia results in ischemic brain damage. In addition, although reperfusion therapy is essential to restore blood flow, it often exacerbates brain damage by augmenting a series of pathological processes, such as oxidative stress, inflammation, and blood-brain barrier disruption (Zhang et al., 2024). Thus, developing strategies for preventing and minimizing brain I/R injury is urgent. Angiogenesis is a process of generating new blood vessels from existing vessels. By improving collateral circulation, pre-ischemic angiogenesis may reduce brain I/R injury. On the other hand, post-ischemic angiogenesis is a critical mechanism to promote functional recovery. It not only increases blood supply to the damaged brain tissue but also provides a scaffold for neurogenesis (Arenillas et al., 2007; Hatakeyama et al., 2020; Hu et al., 2024). Among several growth factors, the VEGF signaling pathway appears to play a key role in cerebral angiogenesis under basal conditions. Furthermore, recent studies indicate that hypoxia-inducible factor 1 (HIF-1) and its target VEGF upregulate and are significantly involved in post-ischemic angiogenesis and neurogenesis (Shimotake et al., 2010; Lee et al., 2022). Interestingly, TGFβ signaling also upregulates VEGF to stimulate angiogenesis (Nakagawa et al., 2004).

Alcohol consumption, a modifiable lifestyle factor, has complex effects on stroke risk and recovery. Multiple meta-analyses suggest that alcohol consumption has a J-shaped relationship with ischemic stroke (Corrao et al., 2004; Patra et al., 2010; Zhang et al., 2014; Larsson et al., 2016). These studies indicate that light-to-moderate alcohol consumption lowers the risk of ischemic stroke, reduces infarct volume and mortality, and improves functional outcomes from ischemic stroke. On the other hand, HAC increases the incidence of ischemic stroke and worsens the prognosis of ischemic stroke (Chung et al., 2023). Consistently, we found that LAC protects against cerebral I/R injury, whereas HAC exacerbates it in male rodent models. In addition, LAC upregulated VEGF-A and VEGFR2 and promoted cerebral angiogenesis under basal conditions and following ischemic stroke. Furthermore, LAC stabilized the BBB and mitigated post-stroke inflammation, while HAC exacerbated these pathophysiological processes (Xu et al., 2019; Li C. et al., 2021; Li J. et al., 2021).

A previous study found that women aged 35-64 are nearly three times more likely than men to have a stroke (Towfighi et al., 2011). Moreover, women with strokes have a lower survival rate compared to men. Among the survivors, women often suffer a worse scenario, including slower recovery and greater severity of disability (Towfighi et al., 2011). Despite these disparities, much preclinical research has historically focused on male subjects (Yoon et al., 2014). The variability induced by hormonal changes in females has often been cited as challenging to experimental reproducibility, contributing to a historical imbalance in research focus between males and females (Clayton, 2016). However, focusing solely on general findings neglects critical sex-specific differences in stroke pathophysiology and recovery, particularly among women who consume alcohol. This oversight leaves a significant gap in our understanding of stroke in this population. Therefore, the goal of this study is to determine the influence of chronic alcohol consumption on cerebra I/R injury in female mice. To elucidate the potential mechanism for the altered cerebra I/R injury, we specifically focused on baseline neurological function and cerebral angiogenesis and post-ischemic cerebral angiogenesis, BBB integrity, and inflammation.

## Materials and methods

### Animal models

All procedures and protocols were approved by the Institutional Animal Care and Use Committee (IACUC) at Louisiana State University (LSU) Health Shreveport and conducted in accordance with the National Institutes of Health Guide for the Care and Use of Laboratory Animals and ARRIVE guidelines. Forty-five female C57BL/6J mice (20-25 g) (4 months) were randomly assigned to three groups: 0.7 g/kg ethanol (LAC group, *n* = 15), 2.8 g/kg ethanol (HAC group, *n* = 15), or water (control group, *n* = 15), and gavage fed with ethanol or volume-matched water once a day for 8 weeks. At the end of 8 weeks, blood pressure and heart rate were measured using a non-invasive tail-cuff system as described previously (Xu et al., 2019). Five mice from each group were euthanized to measure the protein expression of CD31, VEGF-A, VEGFR2, TGF-β, and TGF-βR2. Five mice from each group were euthanized to assess baseline cerebral angiogenesis. The remaining five mice in each group were subjected to transient focal cerebral ischemia to evaluate cerebral I/R injury and post-ischemic cerebral angiogenesis, BBB disruption, neutrophil infiltration, and microglial activation. For euthanasia, the mice were anesthetized with 5% isoflurane in a gas mixture of 30% oxygen and 70% nitrogen via a chamber of the vaporizer for 5 min until the pedal withdrawal reflex disappeared. Subsequently, the mice were subjected to open-chest exsanguination to confirm death.

### Blood alcohol concentration measurement

Blood alcohol concentration was measured from four mice in each group using an Ethanol Assay Kit (ab65343, Abcam) at 15, 30, and 60 min following gavage feeding during the 1*^st^* week. The measurements were performed according to the manufacturer’s instructions. Each mouse only underwent blood collection once from the tail vein.

### Complete blood count analysis

Eight mice from each group were subjected to CBC analysis before and after the 8-week feeding period. The tail was cut under minimal distress, and blood from the tail vein was drawn into heparin-coated capillary tubes. The samples were then transferred into tubes containing EDTA, mixed gently to prevent clotting, and immediately analyzed for complete blood counts (CBC) using an automated hemocytometer (Oxford Science -GENESIS™, Oxford, CT, United States).

### Open field test

Ten mice from each group were subjected to the open field test to assess spontaneous motor activity and anxiety-like behavior at the end of the 8-week feeding period. After habituation to the testing room, mice were placed into a square open field chamber (40 cm L × 40 cm W × 30 cm H) (AccuScan Instruments, Erie, PA, United States). Mice were allowed to explore freely for 30 min, and their movements within the chambers were recorded. The total distance traveled, and the time spent in the inner (center) versus outer (near the wall) zone, a measure of anxiety, were analyzed using the Top Scan Lite-Top View Behavior Analyzing System (Noldus Information Technology, Wageningen, Gelderland, Netherlands).

### Assessment of cognitive function

Ten mice from each group were acclimated to the testing environment following 8 weeks of gavage feeding. Behavioral assessment was conducted in a T-maze under uniform lighting and with minimal sensory cues. The maze was cleaned and disinfected between each mouse to ensure consistent test conditions. During each trial, a mouse was placed in the start arm facing the experimenter, and a temporary blockage was placed at the choice point where the maze’s arms diverged. The blockade was removed after 2–3 s, allowing the mouse to choose between the left or right arms. The initial choice was recorded, and the mouse was held in that arm for 30 s before being returned to the start arm. A second choice was then recorded, allowing the mouse to choose between the left or right arms. Each mouse underwent five trials per day for 3 consecutive days, resulting in 15 total trials per animal. Cognitive performance was inferred from alternation rates (whether the second choice differed from the first) and side preference patterns. These metrics were used to assess the potential impact of chronic alcohol consumption on spatial learning and decision-making.

### Western blot analysis

The cerebral cortex was homogenized in ice-cold lysis buffer (150 mmol/L NaCl, 50 mmol/L Tris-HCl, 10 mmol/l EDTA, 0.1% Tween-20, 1% Triton, 0.1% mercaptoethanol, 0.1 mmol/l phenylmethylsulfonyl fluoride, 5 μg/mL leupeptin, and 5 μg/mL aprotinin, pH 7.4). The homogenates were centrifuged at 12,000 × g for 20 min at 4°C, and the protein concentration in the supernatants was determined using the Bradford protein assay (Bio-Rad, CA, United States). Thirty micrograms of total protein were loaded per well for SDS-polyacrylamide gel electrophoresis (SDS-PAGE) on a 10% gel. Following electrophoresis, proteins were transferred to polyvinylidene difluoride (PVDF) membranes and immunoblotted with primary antibodies against CD31 (RRID: AB 2161028; R&D Systems), TGF-β (RRID: AB 1567351; Santa Cruz Biotechnology), TGF-βR2 (RRID: AB 628347; Santa Cruz Biotechnology), VEGF-A (RRID: AB 2212642; Abcam), and VEGFR2 (RRID: AB2212507; Cell Signaling). Secondary antibodies from Invitrogen (Peroxidase-AffiniPure goat anti-rabbit IgG, anti-mouse IgG, or Peroxidase-AffiniPure donkey anti-goat IgG) were then applied, and protein bands were visualized using an enhanced chemiluminescence (ECL) kit (Pierce Chemical, IL). Band densities were quantified using the Image J software (version 1.54f), and protein expression levels were normalized to GAPDH, with results expressed as fold changes relative to the control group.

### Transient focal cerebral ischemia

To induce transient focal cerebral ischemia, unilateral MCAO was performed for 60 min as described previously (Li et al., 2024). Ethanol was not given on the day of and after MCAO to eliminate the potential acute effect of alcohol. Mice were anesthetized with isoflurane (5% for induction and 1.5% for maintenance) in a gas mixture of 30% oxygen and 70% nitrogen, with body temperature maintained using a temperature-controlled heating pad. The right common carotid artery (CCA) and external carotid artery (ECA) were exposed and ligated. A silicon rubber-coated monofilament (Doccol Corporation, Sharon, MA, United States) was inserted into the right ECA and advanced into the right internal carotid artery (ICA) until it reached the MCA and anterior cerebral artery (ACA) bifurcation. The size of the monofilament was selected based on the body weight of the mouse. After 60 min of occlusion, the monofilament was carefully removed, and the CCA was reopened to allow reperfusion. Seventy-two hours after reperfusion, the mice were euthanized to evaluate infarct volume and post-ischemic BBB disruption, angiogenesis, and inflammation.

### Assessment of post-ischemic sensorimotor deficits

Before euthanasia, a 24-point scoring system was used to evaluate post-ischemic sensorimotor deficits as described previously (Sun et al., 2008; Xu et al., 2019). Six tests (spontaneous activity, symmetry of movement, floor walking, beam walking, symmetry of forelimbs, and climbing wall of wire cage) for motor function and two tests (response to vibrissae touch and reaction to touch on either side of the trunk) for sensory function were graded on a scale of 0-3 each. Neurological scores were assigned as follows: 0, complete deficit; 1, definite deficit with some function; 2, decreased response or mild deficit; 3, no evidence of deficit/symmetrical responses. Thus, a high score represents improved neurological function.

### Immunohistochemistry staining

The mice were perfused transcardially with 25 mL of PBS, followed by 20 mL of 4% paraformaldehyde. The brains were removed and placed in 4% paraformaldehyde for 24 h for fixation. Subsequently, they were subjected to a graded sucrose dehydration process over 72 h, embedded in O.C.T. compound (Fisher Scientific, Hampton, NH, United States), and rapidly frozen in liquid nitrogen for 5 min. Finally, the frozen brains were coronally sectioned at a thickness of 14 μm and mounted onto frost-free microscope slides. Three strategically chosen sections, 1.21 mm rostral to bregma, 0.23 mm, and 1.31 mm caudal to bregma, were selected from each mouse. These sections were rinsed thoroughly with PBS and immersed in a blocking solution containing 0.1% Trypsin and 10% BSA for 1 h. Subsequently, they were incubated with goat anti-CD31 (RRID: AB 2161028, R&D Systems, Minneapolis, MN, United States), rabbit anti-MPO (RRID: AB 307322, Abcam, Cambridge, United Kingdom), or rabbit anti-Iba1 (RRID: AB 839504, Wako Chemicals Inc., Richmond, VA, United States) at 4°C overnight. After three 5-min washes with PBS, these sections were exposed to a 1:200 biotinylated rabbit anti-goat IgG secondary antibody (Vector Labs, Newark, CA, United States) for 1 h. Next, they were treated with either 1:200 streptavidin Alexa Fluor™ 488 conjugate (Thermo Fisher, Waltham, MA, United States) or AlexaFluor 555 donkey anti-rabbit antibody (Santa Cruz Technology, Dallas, TX, United States) at room temperature for 30–60 min. After an additional series of washes, the sections were mounted with a DAPI-containing medium (Vector Labs) and visualized with a 20X objective under a Nikon Eclipse Ts2 fluorescence microscope (Nikon Instruments Inc., Melville, NY, United States). Five images were captured from each region of interest for quantitative analysis, and the quantification was done using NIS-Elements software (BR4.51.00) or Image J software (version 1.52f) as previously reported (Li et al., 2024). We classified microglia as activated if they showed elevated Iba1 expression and had three or fewer processes (Hovens et al., 2014). The data were expressed as fold changes to the control group.

### IgG staining

Three brain sections per mouse, 1.21 mm rostral to bregma, 0.23 mm, and 1.31 mm caudal to bregma, were used for IgG staining. The sections were blocked with a solution containing 0.1% Trypsin and 10% BSA at room temperature for 1 h to minimize non-specific binding. After the blocking step, the sections were incubated with AlexaFluor 488-conjugated goat anti-mouse IgG antibody (1:200, Invitrogen) at room temperature for 3 h. After incubation, the sections were washed thoroughly to remove any unbound antibodies. Staining was visualized using a Nikon Eclipse Ts2 fluorescence microscope (Nikon Instruments Inc., Melville, NY, United States), and the five images from the peri-infarct cortex were processed using the NIS-Elements analysis software (BR4.51.00). The mean fluorescence intensity was measured in regions of the peri-infarct cortex and the corresponding area on the contralateral side. The intensity values were normalized to the contralateral area and expressed as fold changes to the control group.

### Cresyl violet staining

Brain sections, 1.21 mm rostral to bregma, 0.23 mm, and 1.31 mm caudal to bregma, were selected for Cresyl Violet staining to evaluate infarct volume. The sections were cleaned with xylene to remove residual embedding material, dehydrated in ethanol, and stained with a 0.01% Cresyl Violet acetate solution at 60°C for 14 min to highlight the Nissl substance in neurons. After staining, the sections were mounted using a xylene-based mounting medium. Images were captured at 1.0 x magnification using an Olympus microscope, and the extent of ischemic damage was assessed using ImageJ software (version 1.52f). The infarct size (lacking Nissl stain) was calculated by integrating the location of these lesions across the different levels and expressing the total as a percentage of the hemisphere’s volume.

### Statistical analysis

Prism 10 was used for statistical analyses. All data were analyzed with the Shapiro-Wilk test for normality. When the *p*-value was greater than 0.05 in the Shapiro-Wilk test, the data were analyzed with one-way ANOVA followed by Dunnett’s test. When the *p*-value was equal to or less than 0.05 in the Shapiro-Wilk test, the data were analyzed with the Kruskal-Wallis test followed by Dunn’s test. All data were presented as mean ± standard error (SE). The differences are considered statistically significant as the *p*-value is less than 0.05.

## Results

### Physiological parameters

As shown in Table 1, chronic alcohol consumption dose-dependently reduced body weight at the end of the 8-week feeding period. In addition, HAC significantly decreased heart rate [*F*(2, 9) = 5.153; *p* < 0.05]. On the other hand, neither LAC nor HAC altered blood pressure.

**TABLE 1:** Effect of chronic alcohol consumption on body weight, blood pressure, and heart rate.

	Body weight (g)	Systolic BP (mmHg)	Diastolic BP (mmHg)	Heart rate (bpm)
Control	25.8 ± 0.4	122.3 ± 5.7	87.5 ± 1.9	740 ± 40
LAC	23.8 ± 0.3^*^	117.9 ± 4.0	91.1 ± 5.9	707 ± 42
HAC	21.8 ± 0.5^****^	117.1 ± 3.0	81.8 ± 3.6	633 ± 42^*^

The body weight was analyzed using Kruskal-Wallis test with Dunn’s *post hoc*. The heart rate was analyzed using one-way ANOVA with Dunnett’s *post hoc*. All values are means ± SE.

*p < 0.05, and

^****^p < 0.0001 vs. Control.

### Blood alcohol concentration and hematological alteration

**FIGURE 1:**
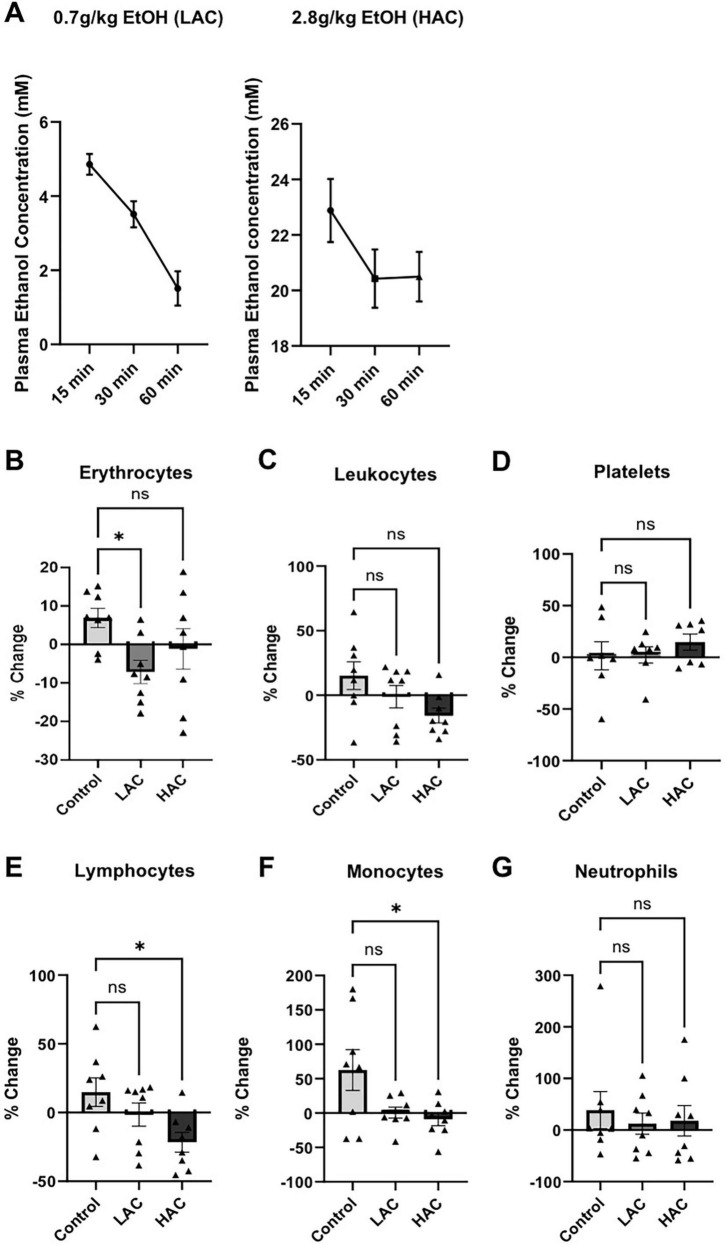
Plasma ethanol level and influence of ethanol on the number of blood cells in female mice. **(A)** Dynamic change of plasma ethanol concentration in the 0.7 g/kg/day and 2.8 g/kg/day ethanol groups at the beginning of an 8-week feeding period. **(B)** Percentage change of erythrocytes at the end of the 8-week feeding period. Analyzed using one-way ANOVA with Dunnett’s *post hoc*. **(C)** Percentage change of leukocytes at the end of the 8-week feeding period. Analyzed using Kruskal-Wallis test with Dunn’s *post hoc*. **(D)** Percentage change of platelets at the end of the 8-week feeding period. Analyzed using Kruskal-Wallis test with Dunn’s *post hoc*. **(E)** Percentage change of lymphocytes at the end of the 8-week feeding period. Analyzed using Kruskal-Wallis test with Dunn’s *post hoc*. **(F)** Percentage change of monocytes at the end of the 8-week feeding period. Analyzed using one-way ANOVA with Dunnett’s *post hoc*. **(G)** Percentage change of neutrophils at the end of the 8-week feeding period. Analyzed using Kruskal-Wallis test with Dunn’s *post hoc*. All values are means ± SE. *P < 0.05 vs. Control.

Plasma alcohol concentration was measured 15, 30, and 60 min after gavage feeding with ethanol. As shown in Figure 1A, the concentration peaked at 15 min, 4.8 ± 0.3mM in the 0.7g/kg ethanol group and 22.9 ± 1.1mM in the 2.8 g/kg ethanol group, and then gradually reduced in the 0.7g/kg ethanol group. In contrast, it remained high at about 20 mM at 30 and 60 min in the 2.8g/kg ethanol group.

To determine the influence of chronic alcohol consumption on the number of blood cells, CBC was performed before and at the end of the 8-week feeding period. There was no significant difference in blood cells among the three groups before the feeding. However, erythrocytes significantly decreased [*F*(2, 21) = 3.483; *p* < 0.05] in the LAC group (Figures 1B). Leukocytes tended to decrease in the HAC group. However, the decrease did not reach statistical significance (*p* = 0.0678) (Figure 1C). Platelets did not alter in either the LAC or HAC group (Figure 1D). Leukocyte subtype count indicates that HAC significantly reduced lymphocytes and monocytes [*F*(2, 21) = 4.394; *p* < 0.05] and had no effect on neutrophils (Figures 1E-G).

### Behavioral function

**FIGURE 2:**
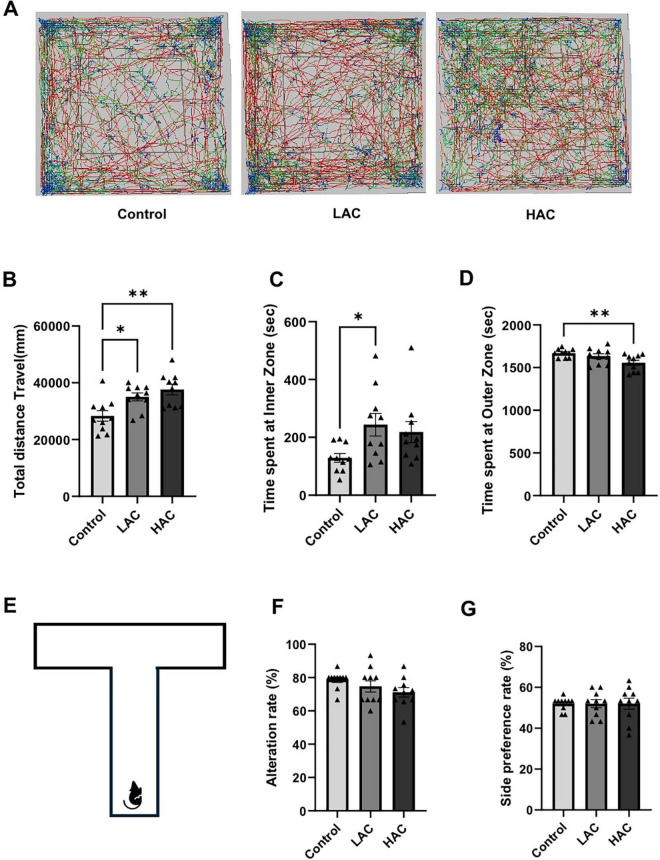
Influence of ethanol on neurological function in female mice. **(A)** Representative tracking plots from the open field test. **(B)** Total distance travel. Analyzed using one-way ANOVA with Dunnett’s *post hoc*. **(C)** Time spent at inner zone. Analyzed using Kruskal-Wallis test with Dunn’s *post hoc*. **(D)** Time spent at outer zone. Analyzed using one-way ANOVA with Dunnett’s *post hoc*. **(E)** Schematic of the T-maze used to evaluate working memory and spatial preference. **(F)** Alteration rate. Analyzed using Kruskal-Wallis test with Dunn’s *post hoc*. **(G)** Side preference. Analyzed using Kruskal-Wallis test with Dunn’s *post hoc*. All values are means ± SE. *P < 0.05, **p < 0.01 vs. Control.

The open field and T-maze tests were conducted at the end of the 8-week feeding period to evaluate the influence of chronic alcohol consumption on neurological function. In the open field test, LAC and HAC increased the total distance traveled [*F*(2, 27) = 7.786; *p* < 0.005], indicating that chronic alcohol consumption promotes spontaneous motor activity (Figures 2A,B). In addition, LAC increased the time spent in the inner zone, and HAC decreased the time spent in the outer zone of the chamber [*F*(2, 27) = 5.171; *p* < 0.05] (Figures 2C,D). Thus, both LAC and HAC tended to reduce anxiety or thigmotactic behavior. In the T-maze test, neither LAC nor HAC changed the alternation and side preference rates, suggesting that chronic alcohol consumption does not affect working memory and spatial preference (Figures 2E-G).

### Cerebral angiogenesis under baseline conditions

**FIGURE 3:**
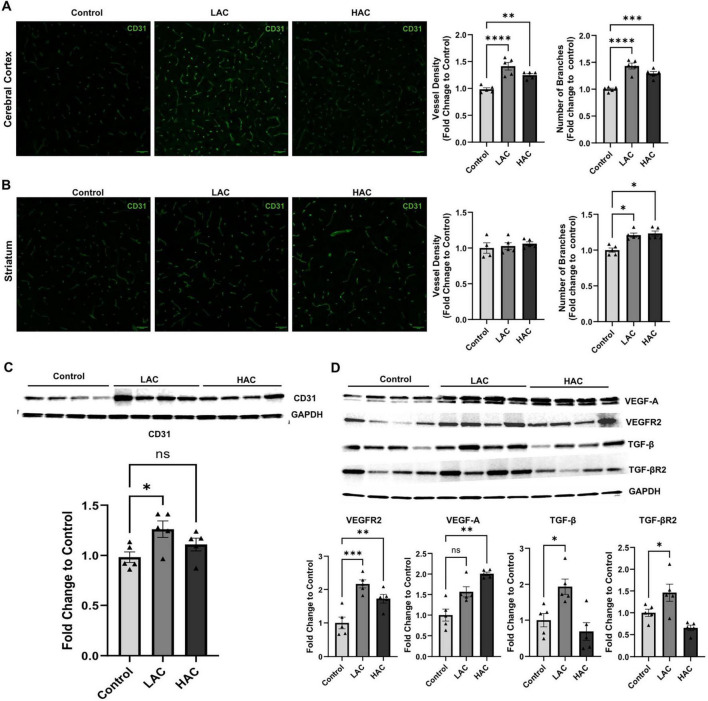
Influence of ethanol on cerebral angiogenesis and growth factors and their receptors in the cerebral cortex of female mice under baseline conditions. **(A)** Representative CD31 staining (left) (scale bar = 100 μm) in the cortex and quantification of vascular density and branch number (right). Analyzed using one-way ANOVA with Dunnett’s *post hoc*. **(B)** Representative CD31 staining (left) (scale bar = 100 μm) in the striatum and quantification of vascular density and branch number (right). Analyzed using Kruskal-Wallis test with Dunn’s *post hoc*. **(C)** Representative Western blots of CD31 (upper) and quantification of CD31 expression (lower) in the cerebral cortex. Analyzed using one-way ANOVA with Dunnett’s *post hoc*. **(D)** Representative Western blots of VEGF-A, VEGFR2, VEGF-A, TGF-β, and TGF-βR2 (upper) and quantification of their expression (lower) in the cerebral cortex. VEGF-A was analyzed using Kruskal-Wallis test with Dunn’s *post hoc*. VEGFR2, VEGF-A, TGF-β, and TGF-βR2 were analyzed using one-way ANOVA with Dunnett’s *post hoc*. All values are means ± SE. *P < 0.05, **p < 0.01, ***p < 0.001, ****p < 0.0001 vs. Control.

Cerebral angiogenesis was determined by vessel density and number of branches via CD31 staining. As shown in Figure 3, vessel density [*F*(2, 12) = 20.85; *p* < 0.0005] and the number of branches [*F*(2, 12) = 29.37; *p* < 0.0001] in the cerebral cortex significantly increased in the LAC and HAC groups compared to the control group. In addition, the number of branches in the striatum also increased in the LAC and HAC groups (Figures 3A,B). Moreover, LAC upregulated CD31 in the cerebral cortex [*F* (2, 12) = 4.366; *p* < 0.05]. Interestingly, although HAC also tended to increase CD31 protein expression, the increase did not reach statistical significance (Figure 3C). On the other hand, both LAC and HAC upregulated VEGFR2 [*F*(2, 12) = 16.72; *p* < 0.0005]. LAC further upregulated TGF-β [*F*(2, 12) = 8.996; *p* < 0.005] and TGF-βR2 [*F*(2, 12) = 9.784; *p* < 0.005] and HAC further upregulated VEGF-A in the cerebral cortex (Figure 3D).

### Cerebral I/R injury and post-ischemic BBB disruption, and cerebral angiogenesis

**FIGURE 4:**
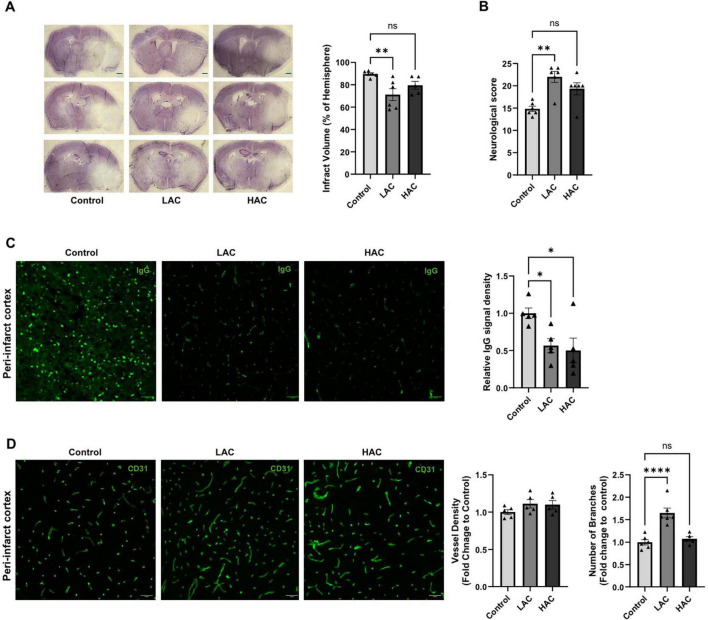
Influence of ethanol on cerebral I/R injury and post-ischemic angiogenesis in female mice. **(A)** Representative cresyl violet staining (left) (Scale bar = 100 μm) and quantification of total infarct volume (right). Analyzed using one-way ANOVA with Dunnett’s *post hoc*. **(B)** Neurological score. Analyzed using Kruskal-Wallis test with Dunn’s *post hoc*. **(C)** Representative IgG staining in the peri-infarct cortex (left) (Scale bar = 100 μm) and quantification of IgG leakage (right). Analyzed using one-way ANOVA with Dunnett’s *post hoc*. **(D)** Representative CD31 staining (left) (scale bar = 100 μm) in the peri-infarct cortex and quantification of vascular density and branch number (right). Analyzed using one-way ANOVA with Dunnett’s *post hoc*. All values are means ± SE. *p < 0.05, **p < 0.01, and ****p < 0.0001 vs. Control.

Cerebral I/R injury, post-ischemic BBB disruption, and post-ischemic cerebral angiogenesis were evaluated at 72 h of reperfusion. As shown in Figure 4A, LAC significantly reduced infarct volume [*F*(2, 14) = 6.383; *p* < 0.05]. In addition, LAC improved post-ischemic sensorimotor function (Figure 4B). Interestingly, both LAC and HAC reduced BBB disruption [*F*(2, 12) = 5.363; *p* < 0.05], indicated by IgG leakage in the peri-infarct cortex (Figure 4C). IgG leakage was not seen in the contralateral corresponding cortex. On the other hand, there was no difference in vessel density among the three groups in the peri-infarct cortex at 72 h of reperfusion. However, the number of branches was significantly greater in the LAC group compared to the control group [*F*(2, 14) = 20.30; *p* < 0.0001] (Figure 4D).

### Post-ischemic neutrophil infiltration and microglial activation

**FIGURE 5:**
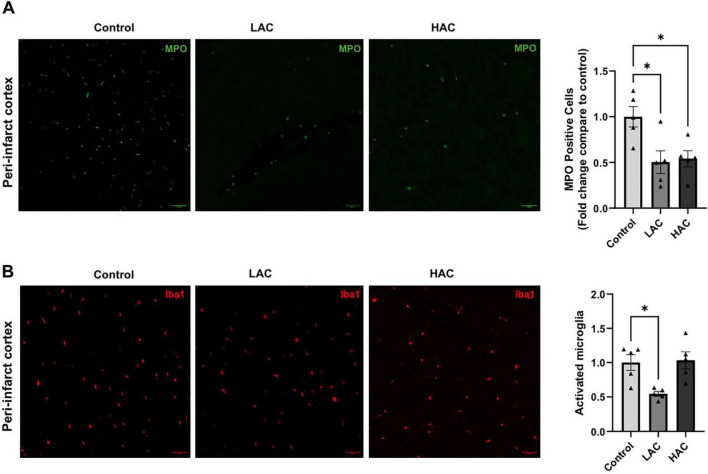
Influence of ethanol on post-ischemic neutrophil infiltration and microglial activation in female mice. **(A)** Representative MPO staining (left) (Scale bar = 100 μm) and quantification of neutrophil infiltration (right) in the peri-infarct cortex. Analyzed using one-way ANOVA with Dunnett’s *post hoc*. **(B)** Representative Iba1 staining (left) (Scale bar = 100 μm) and quantification of microglial activation (right) in the peri-infarct cortex. Analyzed using one-way ANOVA with Dunnett’s *post hoc*. All values are means ± SE. *p < 0.05 vs. Control.

Post-ischemic neutrophil infiltration and microglial activation were evaluated by immunostaining of MPO and Iba1, respectively, in the peri-infarct cortex at 72 h of reperfusion following a 60-min MCAO. As shown in Figure 5A, both LAC and HAC reduced post-ischemic neutrophil infiltration [*F*(2, 12) = 6.57; *p* < 0.05]. Moreover, LAC significantly alleviated microglial activation [*F*(2, 12) = 7.501; *p* < 0.01]. In contrast, HAC did not alter microglial activation (Figure 5B). No neutrophil infiltration or activated microglia were observed in the contralateral corresponding cortex.

## Discussion

The present study investigated the influence of chronic alcohol consumption on cerebral angiogenesis, neurological behavior, and cerebral I/R injury in female mice. There are several new findings. First, LAC reduced erythrocytes, and HAC reduced lymphocytes and monocytes in the peripheral blood under basal conditions. Second, both LAC and HAC increased spontaneous motor activity and tended to reduce anxiety. Third, both LAC and HAC upregulated VEGFR2 in the brain and promoted cerebral angiogenesis. Additionally, LAC upregulated TGF-β and TGF-βR2. Fourth, LAC and HAC reduced early BBB disruption and neutrophil infiltration following transient focal cerebral ischemia. Furthermore, LAC significantly reduced post-ischemic microglial activation and cerebral I/R injury. We speculate that LAC protects the brain against ischemic stroke in females. The neuroprotective benefits may be related to the pro-angiogenic and anti-inflammatory properties of LAC.

In the present study, the peak blood alcohol concentration in the 0.7g/kg ethanol group was about 4.8mM, which usually can be seen in a woman with average body weight (170 lbs) after ingestion of slightly less than one American standard drink (14 grams of ethanol/each). On the other hand, the peak blood alcohol concentration in the 2.8g/kg ethanol group was 22.9 mM, which usually can be seen in a woman with average body weight after ingestion of about four American standard drinks (Abel et al., 1998). In the clinic, light, moderate, and heavy drinking is defined as < 1.2, 2.2, and > 3.5 drinks/day, respectively (Abel et al., 1998). Therefore, the ethanol doses used in the present study represent light and heavy alcohol consumption. In a recent study using male mice, the peak blood alcohol concentration was about 9 and 37 mM in the 0.7 g/kg ethanol group and 2.8 g/kg ethanol group, respectively, higher than those in female mice (Xu et al., 2019). Women metabolize alcohol per unit of lean body mass faster than men (Kwo et al., 1998; Cederbaum, 2012). Thus, our findings are in agreement with what others have observed.

In the present study, LAC significantly reduced infarct volume and improved the functional outcome of transient focal cerebral ischemia. Pre-ischemic cerebral angiogenesis is negatively associated with the infarct size (Li J. et al., 2021). LAC remarkably induced cerebral angiogenesis under basal conditions, especially in the cerebral cortex. It is conceivable that the pro-angiogenic effect of LAC may contribute to its neuroprotective effect against cerebral I/R injury. Interestingly, although HAC also led to pre-ischemic cerebral angiogenesis, it failed to reduce cerebral I/R injury. The mechanisms underlying cerebral I/R injury are complex and involve several interacting elements, such as oxidative stress, apoptosis, BBB disruption, and inflammation. Post-ischemic inflammation, characterized by infiltration of leukocytes and microglial activation, is an essential step in the progression of cerebral I/R injury. Post-ischemic BBB disruption results in brain edema, increases leukocyte infiltration, enhances the hemorrhage transformation incidence, and enlarges infarct volume (Shichita et al., 2012). When neutrophils are depleted from the circulation, the infarct volume is reduced, and cerebral blood flow is improved during the reperfusion period (El Amki et al., 2020). Moreover, activated microglia directly contribute to brain I/R injury by phagocytosis and producing inflammatory and cytotoxic mediators (Xiong et al., 2016). LAC reduced post-ischemic BBB disruption, neutrophil infiltration, and microglial activation. Thus, it is conceivable that the neuroprotective effect of LAC may also be related to its anti-inflammatory properties. On the other hand, although HAC reduced BBB disruption and neutrophil infiltration, it did not affect microglial activation. The difference in the post-ischemic inflammation between LAC and HAC may contribute to the dose-dependent influence of alcohol on cerebral I/R injury. In addition, the impact of HAC on post-ischemic oxidative stress and apoptosis cannot be ruled out.

In the present study, LAC significantly reduced peripheral erythrocytes but not leukocytes, whereas HAC reduced peripheral lymphocytes and monocytes but not erythrocytes. The mechanism that accounts for the different influences remains unclear. Previous studies have found that alcohol negatively impacts erythrocytes in several ways, including damaging bone marrow, macrocytosis, hemolysis, and a lack of vitamin B12 and/or folate (Mueller and Scheller, 2023). Anemia even at a mild level could be a risk factor for ischemic stroke and lead to a poorer outcome following a stroke (Sico et al., 2013; Heo et al., 2021). Thus, LAC appears as a double-edged sword to ischemic stroke. On the other hand, although HAC reduced peripheral lymphocytes and monocytes, it did not alter the number of neutrophils. Thus, it is conceivable that the inhibitory effect of LAC and HAC on neutrophil infiltration is not because of an alteration in the number of peripheral neutrophils. In addition to neutrophils, monocytes and lymphocytes also migrate to the ischemic area, leading to secondary brain damage during the acute phase of ischemic stroke (Planas, 2018). However, they stimulate and contribute to repair during subacute and chronic phases (ElAli and Jean LeBlanc, 2016). Thus, the precise influence of HAC-induced alteration in peripheral lymphocytes and monocytes on ischemic stroke remains to be determined in the future.

A higher level of pre-ischemic locomotor activity is associated not only with a reduced risk of ischemic stroke but also with better functional outcomes following ischemic stroke (Middleton et al., 2013; Meng et al., 2023). Alcohol dose-dependently increased locomotor activity in the present study. Thus, we cannot rule out the possibility that the reduced cerebral I/R injury in the alcohol-fed mice may be partially related to the possible influence of the increased locomotor activity. In general, alcohol consumption has a negative impact on weight loss (Sayon-Orea et al., 2011). However, both LAC and HAC reduced body weight in the present study. The reason for the discrepancies is not entirely clear but may be related to the type of alcohol, method of alcohol intake, locomotor activity, and sex-specific metabolic differences such as altered energy balance, nutrition absorption, and hormonal differences (Liangpunsakul et al., 2010; Wohlgemuth et al., 2021). A recent study reported a strong correlation between underweight and worse outcomes after an ischemic stroke (Sun et al., 2017). Thus, the negative impact of body weight loss on the cerebral I/R injury seems to be overcome, especially in LAC mice. In the present study, both LAC and HAC tended to reduce anxiety. Anxiety has been linked to an increased risk of ischemic stroke and is also associated with more severe strokes (Lambiase et al., 2014). Therefore, although the present study cannot elucidate the precise mechanism underlying alcohol-induced locomotor activity and reduced anxiety, we suggest that behavior changes during alcohol consumption could influence ischemic stroke.

VEGF-A and its receptor, VEGFR2, are major mediators in regulating physiological angiogenesis. In addition, VEGF-A and VEGFR2 also play a crucial role in pathological angiogenesis, such as tumor angiogenesis and post-ischemic angiogenesis. Previous studies have found that light-to-moderate alcohol consumption stimulates VEGF-mediated angiogenesis in several types of tissues, including myocardium, mammary tumor, and chick chorioallantois membrane (Gu et al., 2001; Li J. et al., 2021). In the present study, VEGFR2 is upregulated in the cerebral cortex of LAC and HAC mice. Consistently, vessel density and number of branches significantly increased in the cerebral cortex of LAC and HAC mice. Thus, it is conceivable that VEGFR2 signaling may be involved in alcohol-induced cerebral angiogenesis. Interestingly, LAC but not HAC also upregulated TGF-β and TGF-βR2. However, TGF-β has both angiogenic and angiostatic properties. It leads to cell cycle arrest and cell growth inhibition via the ALK-5/Smard2/3 pathway in most normal cells. On the other hand, TGF-β stimulates cell cycle proliferation and angiogenesis via the ALK1/Smard1/5/8 signaling in endothelial cells (Lebrin et al., 2004; Zhang et al., 2017). In the present study, the magnitude of angiogenesis in the cerebral cortex appears greater in LAC mice compared to HAC mice, suggesting that TGF-β and TGF-βR2 upregulation may partially contribute to LAC-induced cerebral angiogenesis. Previous studies have shown that alcohol consumption upregulates TGF-β in the brain, and upregulated TGF-β may be related to alcohol-induced neurological dysfunction (Bhowmick et al., 2022). Thus, the precise influence of alcohol consumption on TGF-β in the female brain remains to be determined.

We previously used male mice to study the influence of alcohol consumption (Li C. et al., 2021; Li J. et al., 2021; Li et al., 2024). Several differences are noticed between male and female mice in cerebral angiogenesis and cerebral I/R injury during HAC. First, HAC upregulated VEGF-A and VEGFR2 and promoted cerebral angiogenesis in female mice but not in males. Second, HAC reduced post-ischemic BBB disruption and did not alter cerebral I/R injury in female mice. In contrast, HAC exacerbated the BBB disruption, increased infarct size, and worsened neurological function in male mice. Third, HAC reduced post-ischemic neutrophil infiltration and had no impact on post-ischemic microglial activation in female mice but increased neutrophil infiltration and microglial activation in males. In addition to the direct sex-specific influence, the differences could be due to the indirect sex-specific difference in alcohol metabolism rate, which makes the intake of 2.8 g/kg ethanol represent a different number of drinks in male and female mice. Intaking 2.8 g/kg daily is considered HAC in both male and female mice. Based on the peak blood alcohol concentration, however, 2.8 g/kg ethanol represented more than 7 American standard drinks in male mice but only 4 American standard drinks in female mice. Thus, the differences in the influence of HAC on cerebral angiogenesis and cerebral I/R injury between male and female mice may be partially related to the possible impact of different blood alcohol concentrations.

In summary, the present study is the first to experimentally examine the influence of chronic alcohol consumption on cerebral I/R injury in females. It further investigated the potential mechanisms by measuring peripheral blood cells, cognitive function, locomotor activity, cerebral angiogenesis, growth factors, BBB integrity, and inflammation. Although LAC appears neuroprotective against ischemic stroke in both males and females, HAC may have a different influence in males and females. Therefore, the findings of the present study provide innovative information concerning the impact of chronic alcohol consumption on the brain. To fully understand the effects of chronic alcohol consumption on brain I/R injury in females, further studies using aged subjects are necessary.

## Data Availability

The raw data supporting the conclusions of this article will be made available by the authors, without undue reservation.
