# Supplementary figures and images for: Influence of chronic alcohol consumption on cerebral ischemia/reperfusion injury in female mice
Source: Front Cell Neurosci. 2025 Jun 4;19:1600725. doi: 10.3389/fncel.2025.1600725 (PMC12174416; doi:10.3389/fncel.2025.1600725)

**CD31**


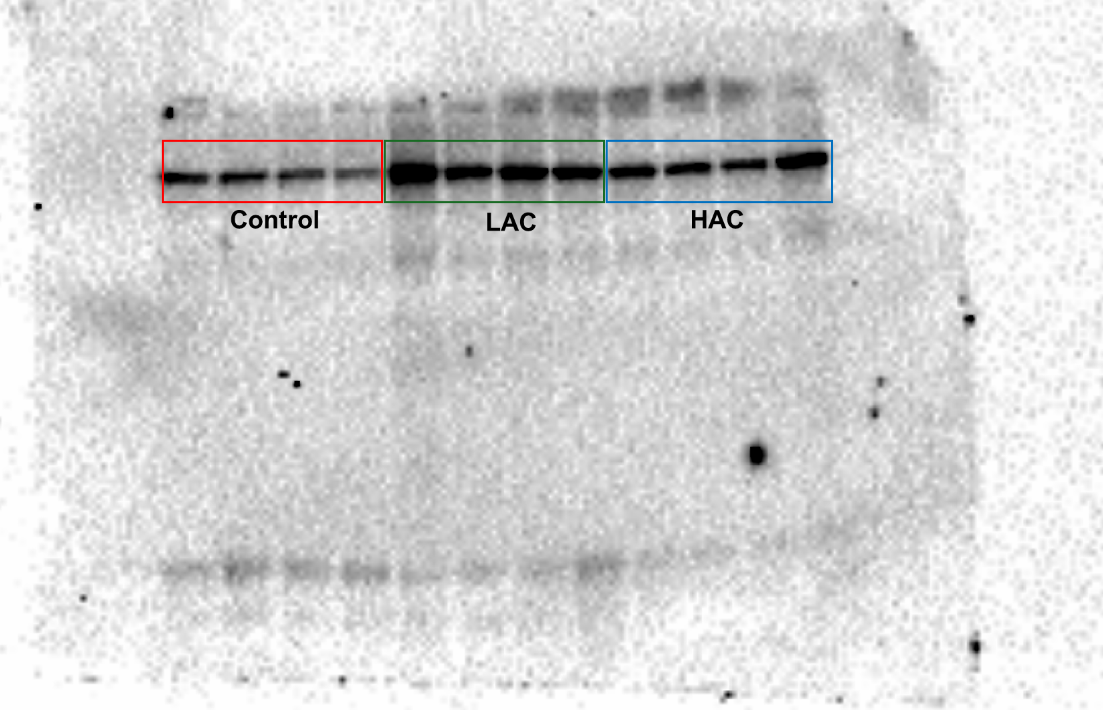


**GAPDH for CD31**


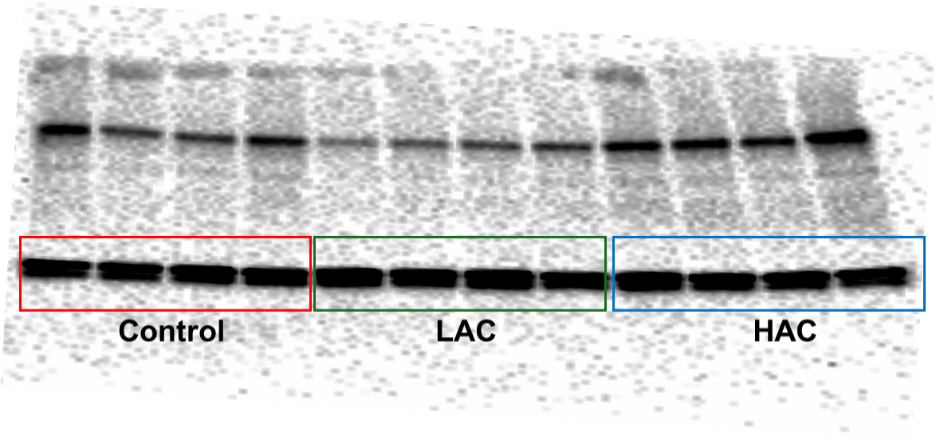


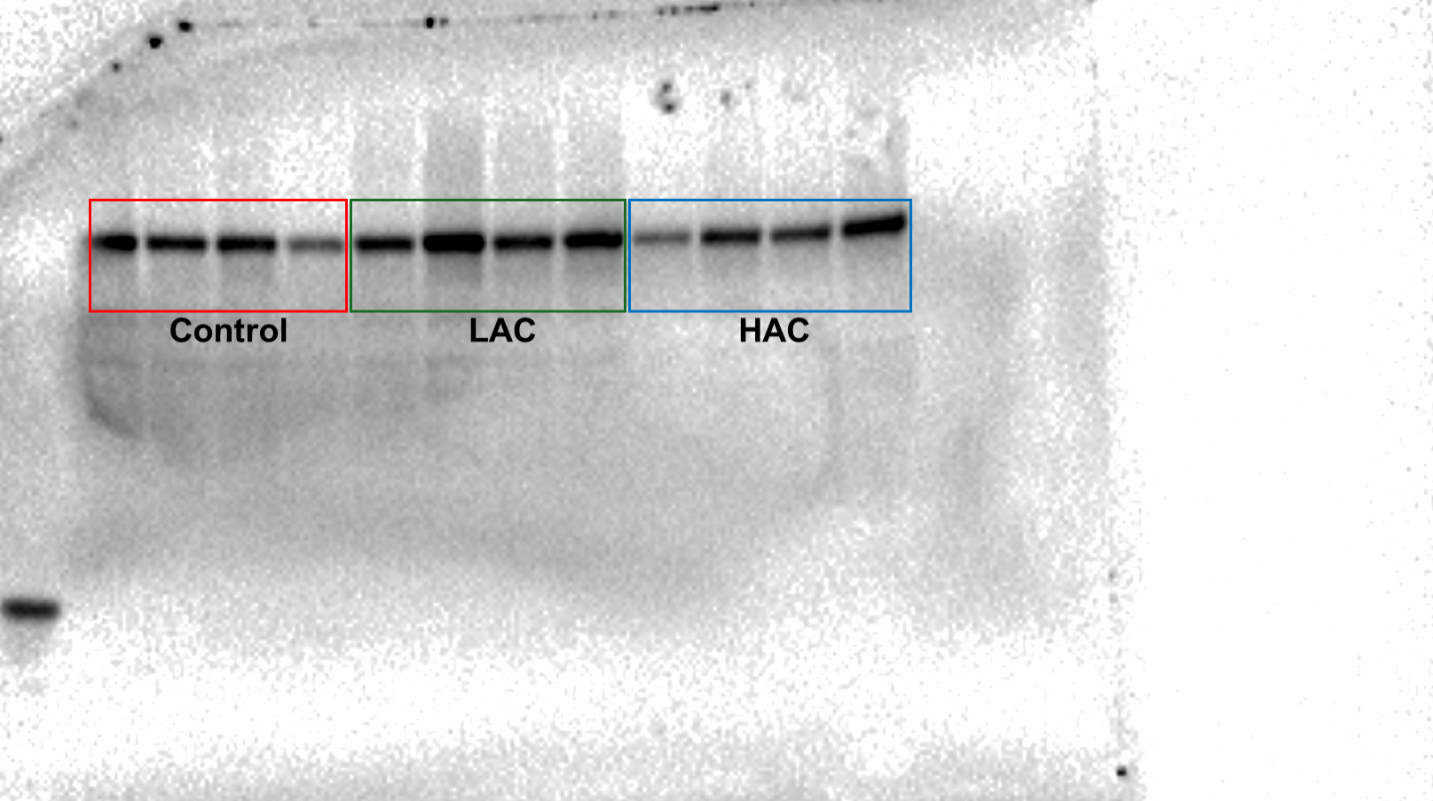


**TGF-β**


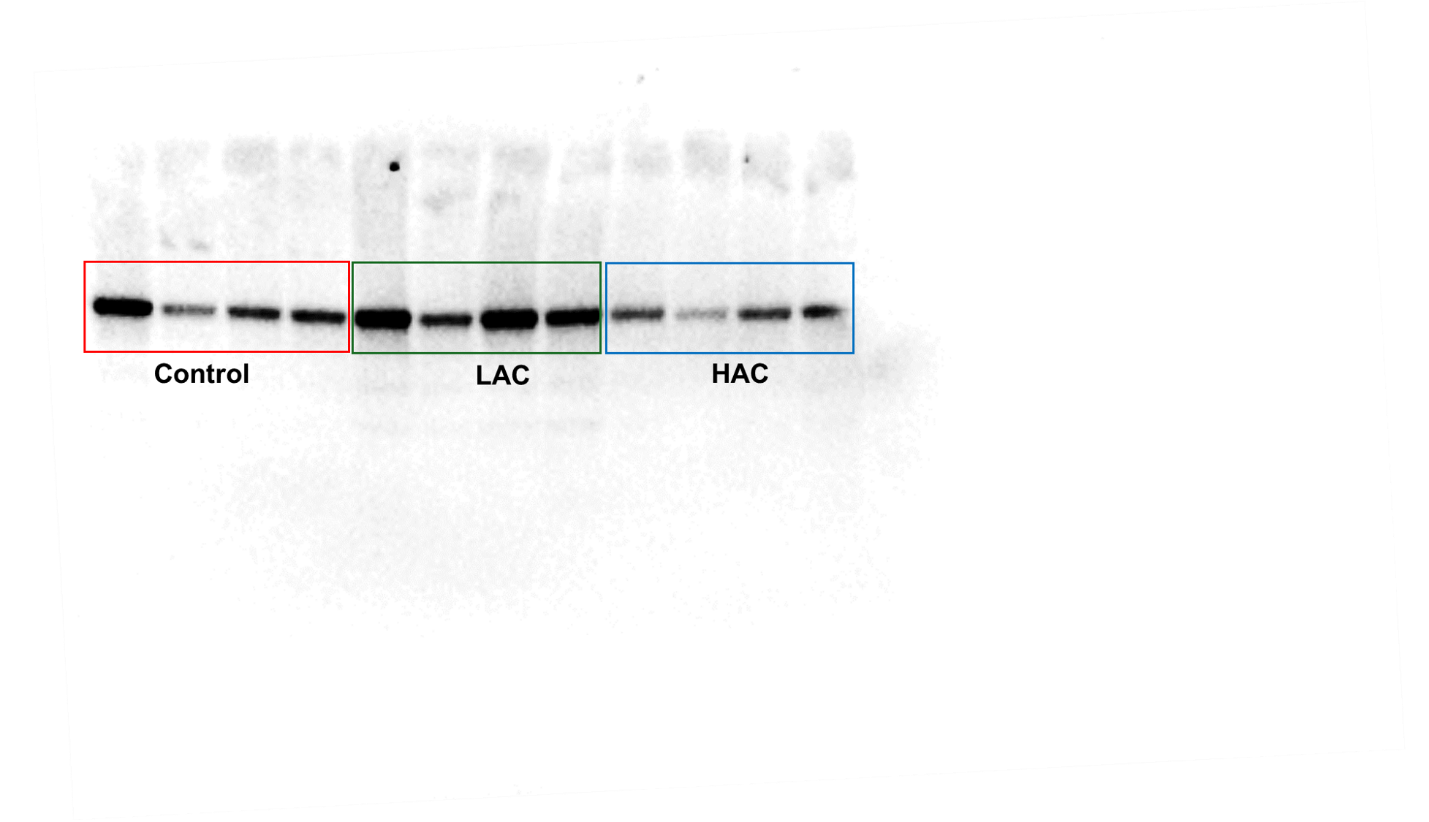


**TGF-βR2**


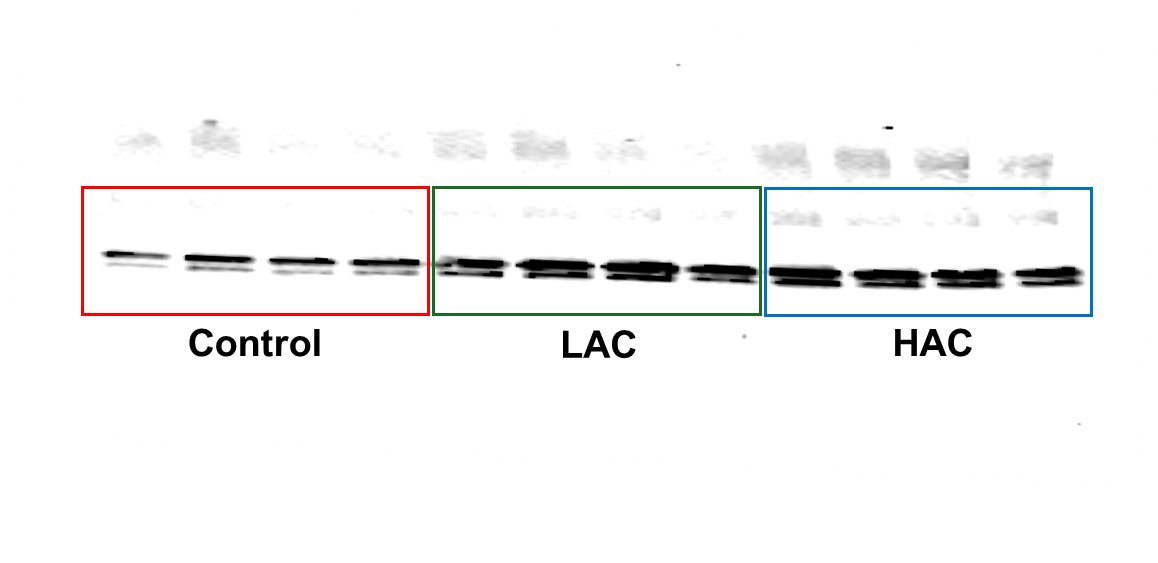


**VEGF-A**

**VEGFR2**


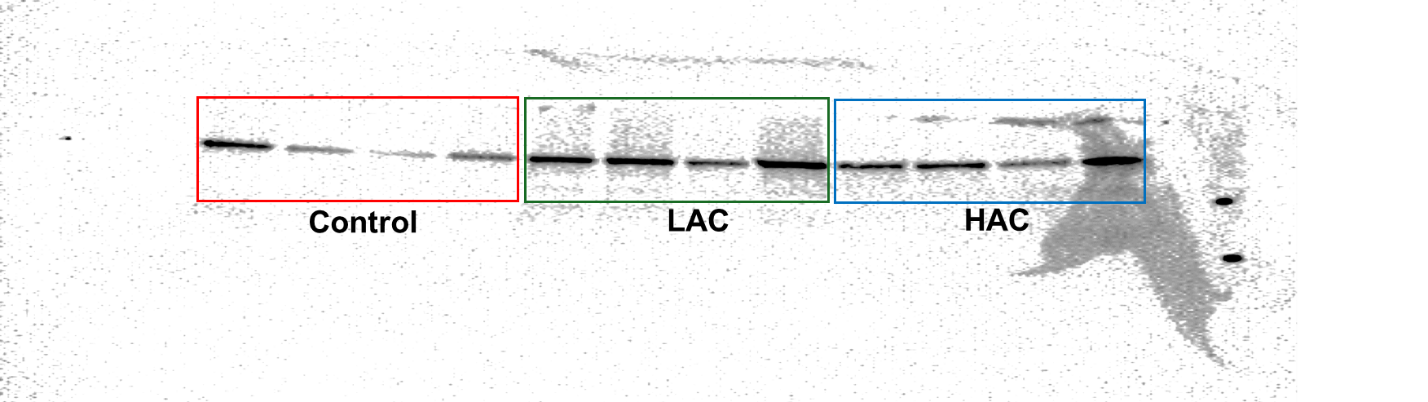


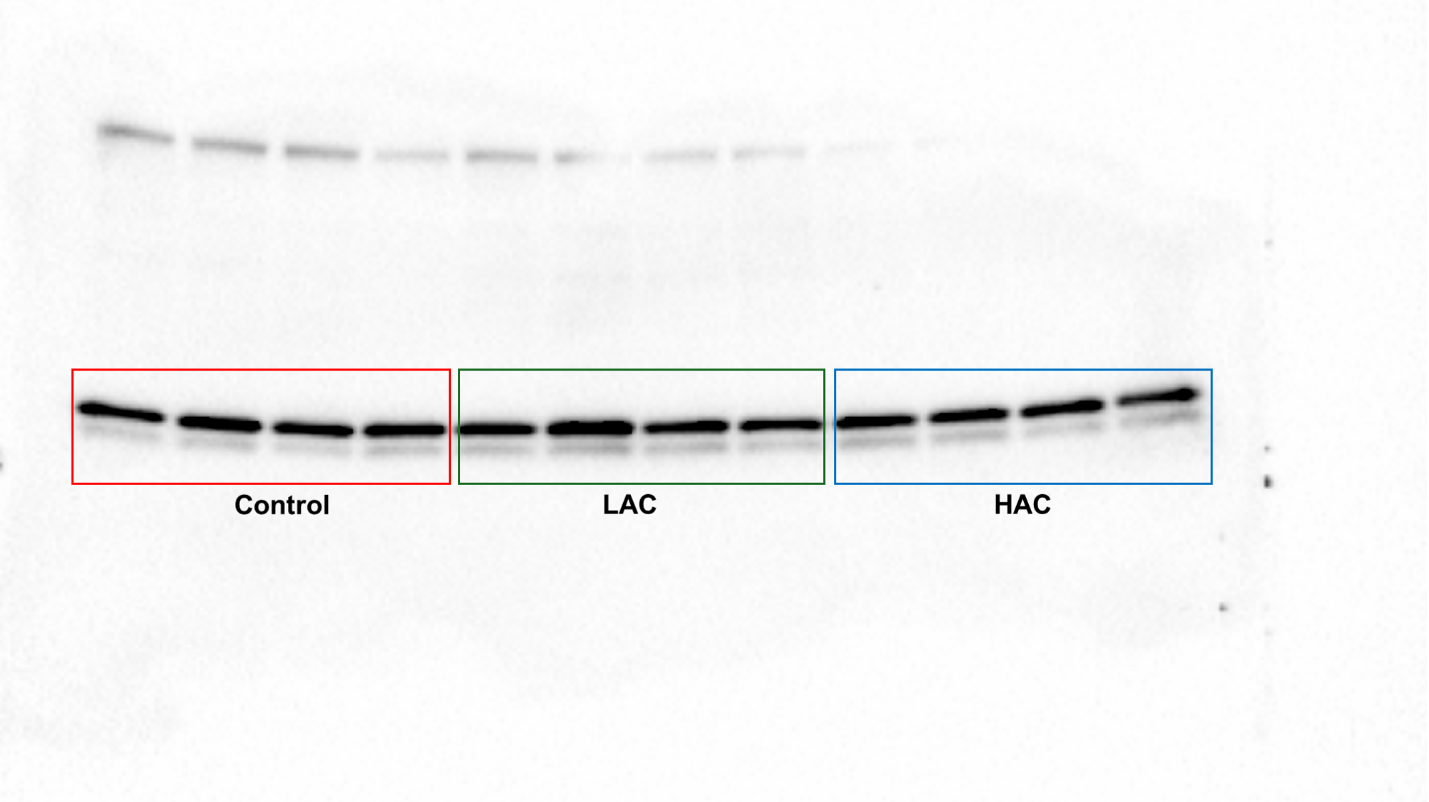


**GAPDH**

Supplement: Supplementary file 1 [file Table_1.docx]
